# Patterns of evolutionary constraints on genes in humans

**DOI:** 10.1186/1471-2148-8-275

**Published:** 2008-10-07

**Authors:** Subhajyoti De, Nuria Lopez-Bigas, Sarah A Teichmann

**Affiliations:** 1 MRC Laboratory of Molecular Biology, Hills Road, Cambridge, CB2 2QH, UK; 2 Research Unit on Biomedical Informatics, Universitat Pompeu Fabra, Dr. Aiguader 88, 08003 Barcelona, Spain

## Abstract

**Background:**

Different regions in a genome evolve at different rates depending on structural and functional constraints. Some genomic regions are highly conserved during metazoan evolution, while other regions may evolve rapidly, either in all species or in a lineage-specific manner. A strong or even moderate change in constraints in functional regions, for example in coding regions, can have significant evolutionary consequences.

**Results:**

Here we discuss a novel framework, 'BaseDiver', to classify groups of genes in humans based on the patterns of evolutionary constraints on polymorphic positions in their coding regions. Comparing the nucleotide-level divergence among mammals with the extent of deviation from the ancestral base in the human lineage, we identify patterns of evolutionary pressure on nonsynonymous base-positions in groups of genes belonging to the same functional category. Focussing on groups of genes in functional categories, we find that transcription factors contain a significant excess of nonsynonymous base-positions that are conserved in other mammals but changed in human, while immunity related genes harbour mutations at base-positions that evolve rapidly in all mammals including humans due to strong preference for advantageous alleles. Genes involved in olfaction also evolve rapidly in all mammals, and in humans this appears to be due to weak negative selection.

**Conclusion:**

While recent studies have identified genes under positive selection in humans, our approach identifies evolutionary constraints on Gene Ontology groups identifying changes in humans relative to some of the other mammals.

## Background

Evolution of eukaryotic genomes is predominantly driven by sequence divergence, insertion, deletion, duplication and recombination, which have introduced enormous diversity into eukaryotic genome architecture. Different regions of a genome evolve at different rates depending on structural and functional constraints [1-3]. Interestingly some regions evolve rapidly only in a given species due to a lineage-specific change in constraints which may affect phenotype [4,5].

The temporal and functional character of evolutionary constraints across genomes, especially in the human lineage, has been of considerable interest recently. Much of this has revolved around non-coding regions. For example, highly conserved elements between human and fugu [6] and between human, mouse and rat [7] have been identified. Pollard et al [4] identified genomic regions that are highly conserved over large evolutionary distance between chicken and chimpanzee but have undergone accelerated evolution specifically in the human lineage.

There have been studies of individual coding regions of the human genome, such as the MHC locus [8], which has a high degree of diversity in the human population. In coding regions, even a moderate change can have a significant consequence, such as the allelic variants of the mu opioid receptor (OPRM1) that is responsible for perception of pain and addiction to drugs in human subjects [9]. A change in constraint on a single locus can influence other genes and have significant phenotypic effects. One classic example is the FOXP2 gene, which is required for brain development associated with motor control of the larynx and mouth, and crucial for developing the articulate speech characteristic of humans [10]. Although the gene is highly conserved within mammals, two of the three amino-acid differences between humans and mice appeared in the human lineage after the separation from the common ancestor with chimpanzee, showing that even small sequence changes can have significant functional consequences [11]. These observations motivated us to focus on the evolutionary patterns in coding regions where most of the base positions are under functional constraints.

Patterns of conservation of genes of different functions are well studied over long evolutionary timescales (reviewed in [12]). For example, different protein families show characteristic patterns of expansion with organismic complexity and genome size [13,14]. Genes involved in core metabolic processes are more conserved [15] while those involved in regulatory [16,17] and peripheral processes like immune response [18] or receptors evolve rapidly. Genes involved in development and organogenesis are fairly conserved in mammals but are quite divergent between vertebrates and invertebrates [12].

At the same time, shorter evolutionary timescales are addressed by comparative genomics and population genetics based studies that scan for genes under recent positive selection in the human genome [19-22]. Several studies have developed methods to incorporate functional information on codon and amino acid substitution to provide a more integrated prediction of selection (reviewed in [23]). Kim and colleagues have studied relationship between positive selection and protein interaction network topology in human and found that proteins located at the periphery of the network and cellular periphery are subject to increased adaptive evolution [24]. Although long-term divergence patterns have been studied in great detail, the patterns of recent evolution of human genes in different functional categories and their effects on selection have not been combined. The classic McDonald-Kreitman test identifies signatures of natural selection by comparing the density of between-species fixed differences (dN/dS) with that of within-species polymorphisms (pN/pS) in coding regions [18]. However, resequencing data for many individuals is needed to do this, and this is still much less common at present than genotyping data. Recently in the targeted analysis of 1% of the human genome as part of the ENCODE Project (pilot phase), the ENCODE consortium compared human polymorphism data (DAF) and comparative genomics data (GERP score), and found significant negative correlation ([25], Supplementary Figure 40). Here we have developed a novel method in order to harness the potential of the flood of human genotyping data as well as the complete mammalian genome sequences.

We call our novel method 'BaseDiver', and use it to identify sets of genes based on patterns of evolutionary constraints on nonsynonymous coding positions in them. Focussing on genes grouped according to function, we ask whether a functional category harbours an excess of nonsynonymous polymorphic coding positions showing specific signatures of constraints, and more interestingly recent changes in evolutionary constraints specifically in the human lineage. Integrating our findings with reports on natural selection on human genes we investigate what evolutionary trends are associated with natural selection on genes, and pinpoint cases where humans accumulate mutations at positions that are conserved in several other mammals.

## Results

**Figure 1:**
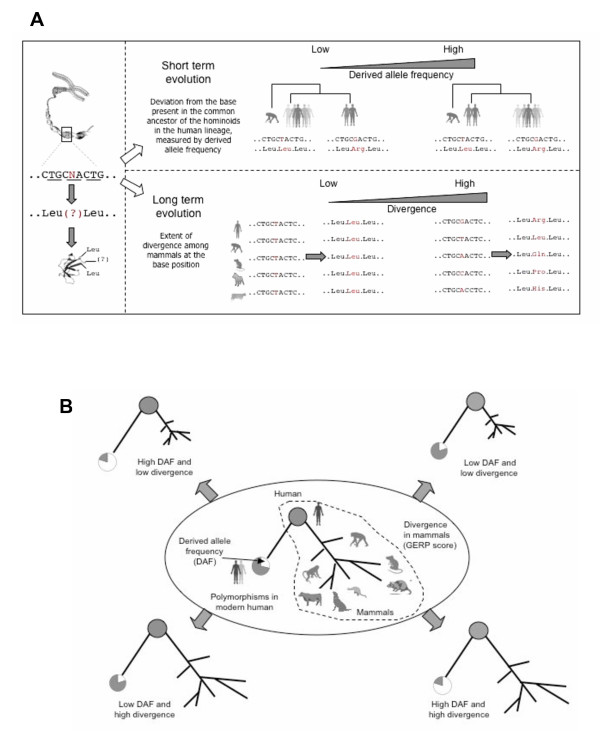
**Patterns of evolutionary constraints in the human genome.** (A) An example is shown where two statistics are studied at a polymorphic coding nucleotide position. The first is short term evolution as derived allele frequency (DAF), reflecting the of drift from the base present in the common ancestor of human and chimpanzee towards a new base in the human lineage. The second is long term evolution (GERP) or the extent of divergence of that base across the mammals considered. Please note that only a few mammals are shown to maintain clarity. (B) At a polymorphic position, integrating short and long term evolution, four basic patterns of evolutionary constraints are possible. Divergence at a base-position among mammals is estimated by the GERP (***G***enome ***E***volutionary ***R***ate ***P***rofiling) score.

In our analysis, we integrate short and long term evolution at nonsynonymous coding base positions to capture patterns of evolutionary constraints at the level of functional categories of genes. At a polymorphic nonsynonymous base position we study two parameters, (a) long-term evolution: the extent of divergence at that base position among mammals, measured by the GERP (***G***enome ***E***volutionary ***R***ate ***P***rofiling) score [26] and (b) short-term evolution: the deviation from the base present in the common ancestor of the hominoids in the human lineage, measured by derived allele frequency (DAF) [27] (Figure 1A). The GERP score reflects the average degree of conservation of a nucleotide position among mammals. The derived allele frequency quantifies the extent humans have diverged away from the base present in the common ancestor of the hominoids. The distributions of DAF and GERP scores for nonsynonymous SNPs are shown in Additional File 1 and 2, respectively.

**Figure 2:**
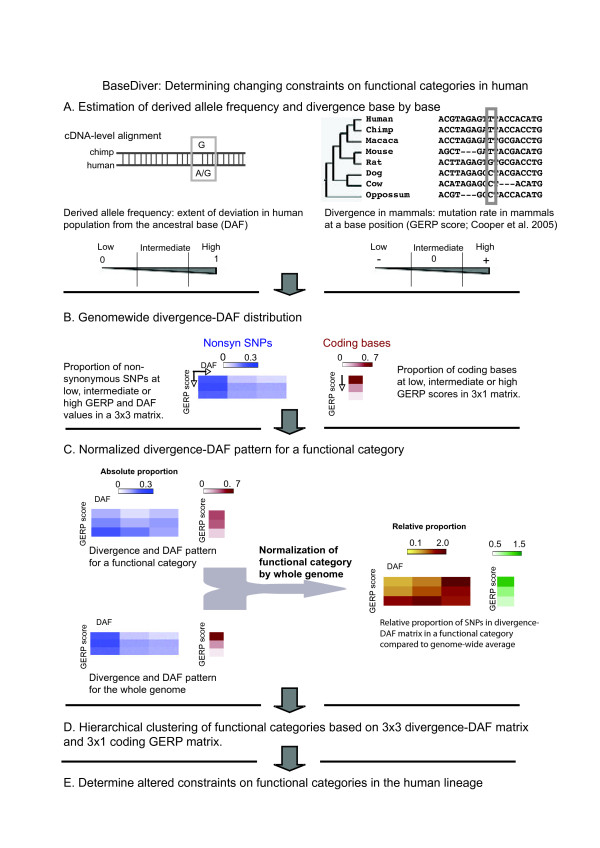
A schematic representation of the BaseDiver algorithm.

Integrating recent (DAF) and long-term (GERP) evolution we categorise the trend at base-position into four basic patterns as shown in Figure 1B, and capture the landscape of evolutionary dynamics at those positions. By considering all nonsynonymous coding base positions of genes in a functional category, we ask whether the category harbours a significant excess of polymorphic nonsynonymous base-positions compared to the background of all nonsynonmous polymorphic positions in genes. If there is a significant difference compared to the whole genome, we categorise it according to the trends shown in Figure 1B. The complete pipeline of this method, that we call BaseDiver, is shown in Figure 2.

Based on the GERP score, we bin the extent of long-term evolution among mammals of a nucleotide position as *conserved (<-1), intermediate (between -1 and 1) *and *divergent (>1)*. At the same time, we group the short-term pattern of evolution measured by DAF as *low *(0.05<DAF<0.33), *intermediate *(0.33≤DAF≤0.67) and *high *(0.95>DAF>0.67). Accordingly we construct a 3 × 3 divergence-DAF matrix and place SNPs in appropriate bins based on their GERP score and derived allele frequency as shown in Figure 2B and Additional File 3.

**Figure 3:**
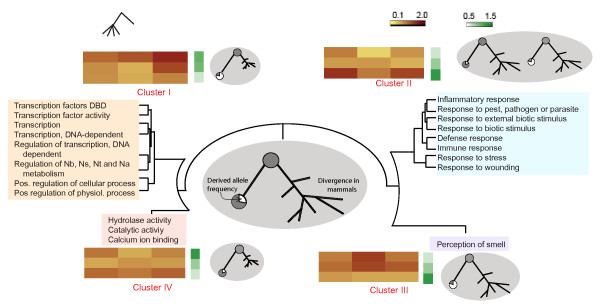
**Patterns of altered evolutionary constraints on different functional categories.** Four clusters of functional categories obtained by hierarchical clustering with characteristic deviations from the genome-wide divergence-DAF distributions are shown. For each cluster, a representative 3 × 3 normalized divergence-DAF matrix (left), 3 × 1 coding GERP matrix (middle) and signature of constraints (right) are provided. Representative matrices are the average of the patterns of all functional categories within the cluster, across all populations. The colour codes of the divergence-DAF matrix and coding GERP matrix are same as in Figure 2C. Signatures of constraints are as in Figure 1. Only functional categories for which signatures are significant in at least three out of four HapMap populations (without Bonferroni correction) are shown. Individual patterns of divergence-DAF matrices for all these functional categories in each population are shown in Additional File 4.

We define the background distribution as the GERP-DAF distribution calculated from all polymorphic nonsynonymous coding base-positions in the human genome and compare the relative abundance of SNPs in different bins of the matrix in a given functional category of genes with the background (see Figure 2C and Methods for details). Thus our null hypothesis is all polymorphic nonsynonymous coding positions in a functional category have a similar GERP-DAF distribution as the background. Our null model is simple and avoided explicit assignment of values for parameters associated with neutral evolution, many of which can be assigned only empirically. We compare this null hypothesis against the alternative hypothesis, that the GERP-DAF distribution of a functional category is different from the background, using a chi-square test (8 degrees of freedom) and assess its significance. Focussing on genes grouped by Gene Ontology (GO) functional categories, we are able to identify functional categories that have a significant excess of polymorphic nonsynonymous base-positions that show specific signatures of evolutionary constraints in the human lineage. We flag a functional category as significant if the divergence-DAF pattern is significantly different from that of the background in at least three out of the four HapMap populations (p-value < 0.05 without Bonferroni correction) [28]. Categories significantly different from the genomic background are shown in Additional File 4. In all cases studied, the signature was significant even after Bonferroni correction in at least one HapMap population. To summarize our observations, we grouped the functional categories by their divergence-DAF pattern using a hierarchical clustering method (please refer to Methods for details). Four clusters emerge, each of which is homogeneous in terms of biological function. The clusters of significant categories are shown in Figure 3, and the characteristics of the clusters are described below. We identified the evolutionary trends associated with each cluster by considering the patterns of constraints on polymorphic base positions from the GERP-DAF distribution as well as the GERP distribution of all coding positions, and information available from the literature, as discussed below.

### BaseDiver Cluster I (Human-specific mutation)

Categories in this cluster are listed in the top left panel of Figure 3. In these categories, the GERP-only distribution of all coding bases of genes in the category exhibits more conservation across mammals than the background of all coding bases in the genome. At the same time, the GERP-DAF distribution shows that there is an excess of nonsynonymous polymorphic positions with high conservation in mammals and high derived allele frequency in humans, suggesting the positions are evolving in a manner unique to humans. All the categories in this cluster revolve around regulation of transcription. While a recent study reports positive selection on transcription factors by considering about 10,000 genes [20], our work at the whole-genome scale shows that transcriptional regulation-related categories are enriched in nonsynonymous positions that have on average high evolutionary conservation in the other mammals but also high derived allele frequency in human. This observation correlates well with the recent finding that primate promoters show accelerated evolution [29] and that human transcription factors have accelerated evolution in terms of expression pattern [30]. Although many such genes or functional categories as a whole may be under positive selection simultaneously in human and other mammals [31], the functional consequences of the mutated base-positions in humans are likely to be unique.

Note that it is less likely that the other mammalian species are polymorphic at same conserved nonsynonymous positions, and it is even more unlikely that the base in the reference sequence corresponds to minor allele. That means our overall interpretation will not be influenced by polymorphism at the same position in other mammalian species.

**Figure 4:**
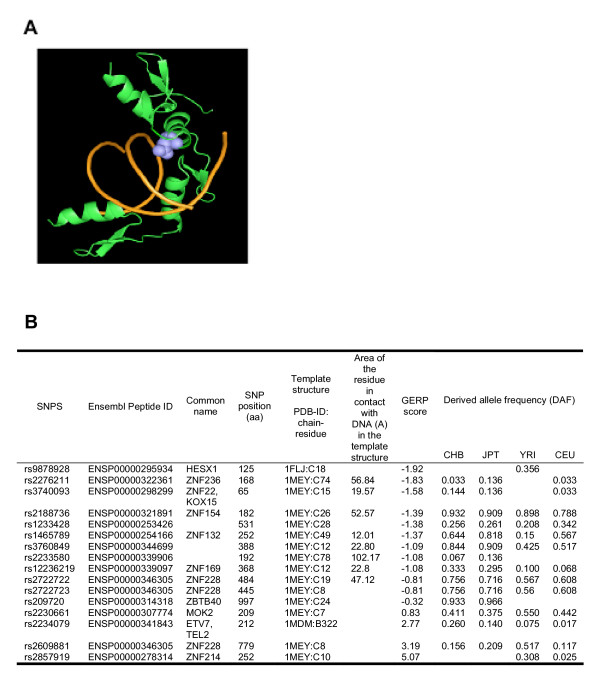
**(A) A cartoon representation of the DNA-binding domain of ZNF228 (green) in contact with DNA (orange).** The nonsynonymous SNP (rs2722722, H484Y) is shown in blue. The complex was modelled based on a homologus structure (1MEY, chain C) taken from the Protein Data Bank (B) Summary of information about nonsynonymous SNPs observed in DNA-binding domains of known and putative transcription factors.

Lineage-specific changes in constraints on functionally important positions may have a significant impact, which may not always be identified by a conventional scan for positive selection. While, for the majority of the proteins, a lack of structural data prevents us from identifying the physicochemical consequences of individual polymorphisms, we can highlight several potentially interesting instances. For example, ZNF228 (ENSP00000346305) a Kruppel-like C2H2 zinc finger transcription factor expressed mainly in heart and haematopoietic tissue has a low (<1) dN/dS ratio with chimpanzee, but harbours three SNPs in the DNA binding domain. One of them (rs2722722: H484Y) is probably in contact with DNA, as an equivalent residue in a homologous structure shares a large interface area with DNA (1MEY:C19; area = 47.1 Å^2^) as shown in Figure 4A. The nucleotide position is fairly conserved in mammals (GERP score: -0.81) but has been subject to selection in humans as apparent from the high derived allele frequency (DAF ∈ 0.5 – 0.75) in all four HapMap populations and high linkage disequilibrium (LD) value (>1.4) in Asian and European populations [22]. A list of nonsynonymous polymorphisms in DNA-binding domains of genes in Cluster I, which can potentially influence DNA-binding properties, is given in Figure 4B. Experimental characterization could reveal the functional consequences of these polymorphisms in humans.

In this analysis we were restricted to using only common SNPs (minor allele frequency > 0.05) from the HapMap project, and this increased the proportion of SNPs with intermediate and high allele frequency in the background distribution as compared to those expected under a neutral model. In addition, several functional categories did not have a sufficient number of SNPs to perform statistical analysis. Therefore it is likely that, when resequencing based dense genotype data is available we will gain increased power to detect many more functional categories that contain an excess of mutations with derived allele frequency higher than the background, and in particular, those with the signature of Cluster I.

### BaseDiver Cluster II (Diversifying selection)

Adaptive processes such as those involved in defense and immune response evolve rapidly in mammals. In these categories, we find an overrepresentation of SNPs with either high or low DAF, while SNPs with intermediate DAF are underrepresented (Figure 3). This means that the adaptive advantage of either allele has lead to its near fixation. One classic example that illustrates this trend within a single locus in two populations is the FY*O allele at the *Duffy *locus that is well known for association with malaria resistance and sickle cell anaemia. The derived allele is near fixation in sub-Saharan Africa but is rare in other parts of the world [32,33]. Large divergence of coding bases in mammals and a strong tendency for rapid fixation of one allele is characteristic of this cluster. Due to the strong signature they leave on the genome, such loci are often identified in scans for positive selection [20-22]. We found several cases where the derived allele frequency differs widely between populations. Some of the genes are associated with immunity. For example, KLRD2 is expressed in NK cells and participate in immune response. A list of the common nonsynonymous SNPs whose DAF between HapMap populations differ by >0.75 are listed in Additional File 5. Function of many of the genes is unknown, and our dataset provides an attractive set of candidates to investigate further.

### BaseDiver Cluster III (Reduced constraints)

In this cluster, which contains genes involved in *perception of smell*, the coding bases evolve rapidly in mammals suggesting reduced constraints on coding base-positions. In addition, there is an excess of nonsynonymous SNPs with intermediate DAF showing relaxed constraints in humans as well. A recent study shows that olfactory receptors are significantly over-represented in human-specific pseudogenes [34], providing further evidence of reduced constraints. Intermediate allele frequency can also arise through balancing selection, where hemizygotes have a selective advantage, thus increasing diversity in a population. However, we infer that an abundance of nonsynonymous SNPs with intermediate DAF, high divergence among mammals and a large number of human pseudogenes points to reduced constraints on these genes in humans. Nevertheless, lineage-specific expansion of these genes makes ortholog detection unreliable and may complicate the patterns of segregation of SNPs therein. So one must be cautious about inferring selection and signatures of constraints on these genes.

### BaseDiver Cluster IV (Strong negative selection)

In this cluster, the coding bases are highly conserved in mammals as shown by the GERP distribution. Polymorphisms occur primarily at bases with a divergent history, and get fixed relatively rapidly, as we observe an excess of SNPs with high GERP score and high or low DAF. Thus, overall there are strong constraints on coding bases both in mammals and in the human lineage. There is only one category in this cluster: *Hydrolase activity*. This cluster is small, because the genomewide constraint on coding bases is generally negative selection, and few categories are constrained significantly beyond this background.

These patterns of evolutionary constraints are consistent across the four HapMap populations (see Additional File 4). To check if the parent-child relationship among GO categories [35,36] bias our observation, we repeated the analysis using the PANTHER classification scheme [37] (see Additional File 6) and found consistent results.

#### Comparison with other studies and integration of evolutionary constraints and natural selection

Several recent studies have used various genomic features to identify genes under positive selection in the human lineage. Here we attempt to gain new insights into patterns of evolutionary constraints associated with selection by integrating findings of BaseDiver with signatures of positive selection on human genes from three large-scale studies that use different genomic features to infer selection and focus on different timescale. Briefly, Nielsen et al [21] used fixed differences between orthologous human and chimpanzee genes and identified selection that is primarily 2–6 million years old. Bustamante et al [20] use a modified McDonald Kreitman test using both fixed differences between human-chimpanzee and polymorphism data within the human lineage to infer strongly selected genes over a similar timeframe. Both these works used re-sequencing of protein-coding regions to identify variation within and between species. Voight et al [22] used linkage disequilibrium (LD) estimated by iHS statistic to identify loci which have been under selection in the last 45,000–70,000 years. Using the HapMap data, they identified signatures of positive selection on one allele over another in recent evolutionary time. In contrast to the previous two methods, the analysis depends on tag-SNPs and does not involve re-sequencing.

These studies use various different approaches to identify signatures of selection. Unlike these approaches, our method does not use a neutral model and therefore is not an explicit test for natural selection. BaseDiver identifies patterns of evolutionary constraints on base-positions polymorphic in a lineage by comparing to a background dataset that is itself under purifying selection (all nsSNPs). This provides insights complementary to those in the previous studies. Here we qualitatively compare our results with the findings of three studies on selection at the level of functional categories. The functional categories in our Clusters I, II and III are repeatedly identified as being under positive selection in previous work (Table 1). By inspecting the difference between the patterns in the three Clusters, our results provide information about the evolution of these categories beyond positive selection, as explained in the following paragraphs. Using BaseDiver we can further differentiate functional categories of genes, which are classified as being under positive selection in the three studies, based on the patterns of evolutionary constraints on the mutated base-positions on genes in those categories.

**Table 1:** BaseDiver distinguishes between evolutionary patterns associated with positive selection.

Evolutionary constraints and natural selection on functional categories	BaseDiver	Nielsen et al. PLoS Biology 2005	Bustamante et al. Nature 2005	Voight et al. PLoS Biology 2006
	• 16, 223 human genes with orthologues in mammals	• 13,731 human genes with orthologs in chimpanzee.	• 11,624 human genes with orthologs in chimpanzee.	• Whole genome analysis covering all genes.
	• Divergence-DAF distribution measuring change in constraints	• dN/dS ratio test for positive selection	• McDonald Kreitman test for positive selection	• iHS statistic estimating linkage disequilibrium
	• 76 Biological processes and 146 Molecular functions-GO classification	• 133 Biological processes – PANTHER classification	• 133 Biological processes and 139 Molecular functions – classification	• 222 nested and overlapping PANTHER classes
**Transcription** **related** **genes**	**Cluster I:**			
	• Transcription factor activity.		• mRNA transcription	• mRNA transcription initiation
	• Transcription factors DBD		• Transcription factor	• Chromatin packaging/remodeling
	• Transcription		• Zinc finger transcription facto	
	• Regulation of transcription		• Nucleoside, nucleotide and nucleic acid metabolism	
	• Regulation of transcription nucleobase, nucleoside, nucleotide and nucleic acid metabolism.		• Homeotic transcription factor	
			• KRAB box transcription factor	
			• Nuclear hormone receptor	
**Immunity** **related** **genes**	**Cluster II:**			
	• Inflammatory response	• Immunity and defense	• Natural killer cell mediated immunity	
	• Response to stress	• T-cell-mediated immunity	• Defence/immunity protein	• MHC-I-mediated Immunity
	• Response to wounding	• Natural killer-cell- mediated immunity	• Immunoglobin receptor family member	• Peroxisome transport
	• Immune response	• Interferon mediated immunity		
	• Defense Response	• B-cell and antibody mediated immunity		
	• Response to pest, pathogen or parasite.			
**Sensory ** **perception** **related ** **genes**	**Cluster III:**			
	• Perception of smell	• Chemosensory perception	• Sensory perception	• Chemosensory perception
		• Olfaction		• Olfaction
				• Other carbohydrate metabolism
				• Electron transport
**Other ** **categories**	**Cluster II:**			• Steroid metabolism
	• Response to biotic stimulus	• Inhibition of apoptosis	• Protein kinase	• Lipid and fatty acid binding
	• Response to external biotic stimulus		• Receptor	• Protein modification
			• Apoptosis	• Vitamin/cofactor transport
				• Phosphate Metabolism

The studies by Bustamante et al. and Voight et al. identify significant signatures of selection on genes involved in transcription (Cluster I) while our findings highlight that these categories harbour an excess of polymorphic bases are likely to be unique to humans. Taken together, our results suggest that, although transcription factors may also be under selection in other primates [31], positive selection on transcription factors has unique consequences in human.

The genes involved in defense and immunity (Cluster II), evolve rapidly under strong selection pressure both within and between species. Previous work (Table 1) shows that these genes are under positive selection between species. We find that these genes diverge quickly in mammals and in human contain an excess of polymorphic loci that show strong preference for one allele over the other, suggesting diversifying selection. Strong selection pressure on the immune system to cope with changing environment and disease, and response to stress, drive the selection on these genes over a broad evolutionary time-spectrum.

Cluster III represents genes involved in perception of smell. We see rapid evolution of these genes in mammals, and our finding of intermediate allele frequency for mutated nonsynonymous bases in these genes in human, suggest that positive selection on these genes is not driven by the same evolutionary forces as in the case of Cluster I & II. Taking our results together with the finding that a large number of remnants of olfactory receptors in the pool of human pseudogenes [34], we conclude that these genes are under reduced constraints in human. However, lineage-specific expansion and copy number variation of gene families involved in olfaction [38] may complicate the orthology detection and influence the signature of selection detected.

While the recent studies including the three mentioned above identify signatures of natural selection on protein-coding genes, our approach adds value by providing information about how instances of positive selection can have different evolutionary footprints, and possibly have different functional implications. The above comparison of different studies highlights that although genes involved in diverse biological processes like transcription, defense and immunity have all been subject to positive selection in the human lineage, our results show that there are differences in the patterns of evolutionary constraints between these functional categories. Some of these changes are unique to humans, and may make a significant contribution towards the difference between us and other mammals.

## Discussion

Genotyping and sequencing of a number of eukaryotic genomes provide us with an opportunity to study the temporal and functional character of evolutionary changes in metazoans. Here we provide a framework for identifying changes in evolutionary constraints on mutated positions in the human genome. Due to lack of SNP data, in the current analysis we captured only higher order patterns at the level of functional categories. But upon availability of resequencing data, using BaseDiver it is possible to achieve higher resolution. In this work we restricted the use of BaseDiver to coding regions only, where most of the base positions are under selection and the effects of hitchhiking are small, it can be used to identify changes in constraints in non-coding regions as well.

Recently outliers of evolutionary patterns like ultra-conserved elements in higher eukaryotes and highly accelerated regions in humans have been identified [4,7]; here we attempt to capture the comprehensive spectrum of evolution of coding regions using BaseDiver. We study the tempo of recent patterns of constraints on human genes grouped by their functional categories and identify which categories are under altered constraints in humans only and which are evolving rapidly in related species. While recent studies have reported several functional categories under positive selection in the human lineage, we go further by characterising the particular evolutionary pattern of altered constraints in each category. Our study uncovers that different functional categories under positive selection during human evolution are under different evolutionary constraints, and some of them (*e.g*. transcription factors) are accumulating an excess of nonsynonymous mutations in humans at positions that are conserved in other mammals.

As Zhang and colleagues report [31], we agree that many genes and functional categories may also be under altered constraints in chimpanzee or other mammals, but the mutated base positions and hence functional consequences are likely to be different from those in human.

Summarizing our observations and putting them in the context of recent work, we find that:

(1) Transcription factors show a significant excess of nonsynonymous positions under accelerated evolution specifically in humans. Changes in evolutionary constraints on some of the loci in transcription factors may have a significant functional consequence, as in the case of ZNF228. Such small-scale changes on transcription factors in turn can introduce large-scale transcriptome-level changes *via *downstream target genes. In 1975, King and Wilson observed that the sequence-level difference between human and chimpanzee is small and proposed that regulatory changes may be important for the difference between human and primates [39]. Consistent with the hypothesis, several recent independent studies including ours show that elevated *cis*- and *trans*-regulatory changes, many of which are unique to humans, may have driven the transcriptome evolution leading to humans [30,40].

(2) Immunity related genes evolve rapidly in mammals including humans. Pressure to survive against pathogens and environmental changes has imposed strong selection on polymorphic positions, driving fixation of one allele over another. Many of the loci showing strong signatures of recent selection in the study by Voight et al. [22] are involved in immune response.

(3) We also find that the genes involved in perception of smell evolve rapidly in all mammals as well as in humans suggesting that their evolution at a rate higher than the neutral rate is not specific to humans. In our BaseDiver analysis we find very few categories (e.g. *Hydrolase activity*) showing signatures of strong constraints. Given that the vast majority of coding regions are under some negative selection, regions under very strong negative selection relative to the genomic background are rare. In addition, these regions are less likely to harbour many polymorphic loci and thus would remain undetected in a sparsely genotyped human genome by our approach. BaseDiver will be more effective in a densely genotyped map of the human genome when it becomes available.

The extent of evolutionary conservation of loci in coding regions can be studied at various levels of granularity when resequencing based polymorphism data will be available for human.

We wish to highlight two points. First, in this study we have grouped genes based on semiautomated Gene Ontology classification, and some of the categories are very broad (e.g. *metabolism *contains genes that can be further sub-classified into finer categories). Therefore, our observations depend on the accuracy with which genes are assigned to GO ontology groups as well as the appropriateness of the ontology itself. Second, while integration of data in a bottom-up approach helps us see overall constraints, imposed by functional categories for example, at certain instances individual loci may be subject to different constraints. For example, categories involved in primary metabolism are under strong negative selection. But the LCT locus, the functionality of which is responsible for lactase persistence, has been under positive selection in the European population [41]. It is likely that when several mutated nonsynonymous coding positions in a functional category are under similar constraints, they cumulatively confer an overall characteristic signature of evolutionary constraints on the functional category.

In this analysis, we were limited to using only the common SNPs (minor allele frequency >0.05) from the HapMap project. Upon availability of re-sequencing based polymorphism data it will be possible to increase the power of our method to detect more subtle patterns. In addition, in a more densely genotyped map of the human (or other species) genome, it will be possible to control the level of granularity using BaseDiver and identify changes in constraints at different levels. While other methods identify signatures of selection in a genome using different genomic features, our method provides a complementary view identifying whether changes are specific to the reference organism. Availability of high quality genome-wide polymorphism data in non-human species will help us in the future to distinguish the patterns observed in human from those in other species.

## Methods

### Mammalian genomes used

In the current analysis, coding sequences from eight mammalian genomes were taken from Ensembl v. 37: Human (*Homo sapiens *– NCBI36), chimpanzee (*Pan troglodytes *– CHIMP1A), monkey (*Macaca mulatta *– MMUL_0_1), mouse (*Mus musculus *– NCBIM34), rat (*Rattus norvegicus *– RGSC3), dog (*Canis familiaris *– BROADD1), cow (*Bos taurus *– Btau_2.0), oppossum (*Monodelphis domestica *– BROAD02). The results were also calculated using Ensembl v. 42 and were consistent with Ensembl v.37.

### Orthology detection

Orthologs of the human genes in these mammals were taken from Ensembl-Compara v. 37 which used the bi-directional best-hit method to identify orthology. We considered only orthologs that were >100 amino acid long aligned over at least 70% of the human protein. The conclusions in this work are consistent when we re-calculated all results using Ensembl v. 42 which uses maximum likelihood phylogenetic trees for orthology detection (data not shown).

### Functional categories

Genes were grouped based on their involvement in Biological Processes and Molecular Functions according to the Gene Ontology classification. If there were at least 100 SNPs in genes in a functional category, the category was considered for BaseDiver analysis. 146 Biological Process and 76 Molecular Function categories met this criterion. The analysis was also performed using functional categories based on the PANTHER classification scheme [37] (see Additional File 6).

### Short-term evolution within human populations

To study the extent of short term evolution at the base level in the human genome, we considered the nonsynonymous coding SNPs with minor allele frequency >0.05 from the HapMap project [28] that has systematically genotyped 269 individuals in total from (i) a Chinese Han population in Beijing (CHB), (ii) a Japanese population from Tokyo (JPT), (iii) an African Yoruba population from Ibadan, Nigeria (YRI) and (iv) a Caucasian European people from Utah (CEU). The CHB and JPT populations are 45 unrelated individuals each, and the YRI and CEU populations are 30 parent-offspring trios. Data were taken from the ENSEMBL repository http://www.ensembl.org. There were about 10,000 SNPs in this dataset.

By restricting ourselves to common nonsynonymous SNPs (minor allele frequency >0.05), we lose information about rare SNPs, but avoid the problem of ascertainment bias that is particularly relevant for these SNPs [42]. In order to verify that the frequencies and coverage of the common SNPs is good, we carried out comparisons with Perlegen and ENCODE data (see Additional File 7).

To identify the ancestral state of the nondsynonymous coding SNPs in human we aligned orthologus DNA sequences based on the corresponding protein sequence alignment between human and chimpanzee, and compared the human SNP with the base at that position in chimpanzee. The allele matching the chimpanzee base was considered as the ancestral allele, and the other allele is taken as the derived allele (Figure 2A). It is possible that (i) a SNP is older than speciation and exists both in human and chimpanzee lineage or (ii) both human and chimpanzee develop SNPs at same position independently. In both these cases ancestral allele determination is difficult taking only chimpanzee base position as a reference, and a three-way analysis including macaque genome can resolve the ambiguity [43].

For several reasons, we chose to determine the ancestral allele by two-way and not three-way comparison. First, limited genotyping found a very small number of polymorphic positions shared between human and chimpanzee (<0.4%) [44]. In nonsynonymous coding positions, which are under constraints, the number of cases, where both human and chimpanzee have accumulated mutations towards the same derived base (thus giving rise to mis-prediction of the ancestral state) would be even lower. Secondly, incorporation of the macaque genome reduces the coverage significantly due to (i) presence of gaps and (ii) different nucleotides at aligned positions, or (iii) simply absence of orthologous genes. Thus, in coding nonsynonymous positions, inclusion of the macaque genome could contribute to a small increase in accuracy (<0.5%) at the cost of a relatively large decrease (>20%) in the size of the dataset we used. Thus we determined the ancestral state using the chimpanzee genome alone.

We were able to identify the ancestral allele identified for ~80% of the SNPs in our initial dataset (7896 SNPs). Ancestral state prediction for ~30% of the SNPs in our dataset was available at from the dbSNP repository ftp://ftp.ncbi.nih.gov/snp/database/b124/mssql/data/SNPAncestralAllele.bcp.gz and ftp://ftp.ncbi.nih.gov/snp/database/b124/mssql/data/Allele.bcp.gz (May'06). We used ancestral allele information provided by dbSNP to benchmark our approach. Agreement between dbSNP and our predictions for the overlapping entries was ~89%. While dbSNP assigns the ancestral state of SNPs from information provided by depositors based on small and large-scale studies, we used rigorous human-chimpanzee coding region alignment, which is highly accurate but can be applied to orthologus coding regions only. Correlation of derived allele frequency for four HapMap populations is shown in Additional File 1.

### Long-term evolution across mammals

To study the nucleotide-level divergence profile of orthologus regions across mammals we used the GERP (***G***enome ***E***volutionary ***R***ate ***P***rofiling) method by Cooper and colleagues [26] (Figure 2A). The GERP score at a base-position quantifies the conservation of a base position across the mammals considered.

DNA level multiple alignment of orthologus coding regions was performed using Dialign [45]. The phylogenetic tree of the mammalian genomes was constructed by eliminating nodes that were not present in our study from the tree provided by Cooper et al http://mendel.stanford.edu/SidowLab/downloads/gerp/index.html. From the tree, the average neutral rate of substitution for the mammalian genomes included in the analysis was taken as 1.93 substitutions per base. Semphy [46] was used by GERP to calculate the observed rate of divergence on a base-by-base basis. The score for evolutionary divergence was calculated as: *GERP score = Observed- Expected Rate*. Around 15000 genes that had orthologs in 4 or more mammalian genomes were subject to GERP analysis. A GERP score could be calculated for 7414 SNPs (92%) in our dataset. GERP score distribution of coding SNP and non-SNP positions are shown in Additional File 2.

SNPs in other species could potentially complicate estimation of the GERP score. Unfortunately, high quality genome-wide polymorphism data is not available for non-human species. On the other hand, patterns of allele frequencies in the HapMap populations show that very few nonsynonymous SNPs have derived allele frequency greater than ancestral allele frequency (see Additional File 1). Hence, in this work we follow an assumption that reference sequences for non-human species always contain the major allele.

### Divergence-DAF joint distribution

Polymorphic loci in our dataset had two statisticss, DAF for the extent of recent evolution, and the GERP score for the extent of long-term evolution. DAFs were binned into three ranges: *low *(DAF: 0.05 – 0.33),*intermediate *(DAF: 0.33 – 0.67) and *high *(DAF: 0.67 – 0.95). GERP scores were binned into *conserved *(GERP score < -1),*intermediate *(GERP score: -1.0 – 1.0) and *divergent *(GERP score: >1.0). Any SNP in the dataset was placed into the appropriate bin of the 3 × 3 'divergence-DAF matrix' according to its GERP score and DAF in a given HapMap population. As an estimate for the background divergence pattern, all coding (synonymous and nonsynonymous) bases were placed in 3 × 1 'coding GERP matrix' which was binned as *conserved *(GERP score < -1),*intermediate *(GERP score: -1.0 – 1.0) and *divergent *(GERP score: >1.0) (Figure 2B). Divergence-diversity matrices for synonymous and nonsynonymous SNP positions are shown in Additional File 3.

We define the background distribution as the GERP-DAF distribution calculated from all polymorphic nonsynonymous coding base-positions in the human genome and compare the relative abundance of SNPs in different bins of the matrix in a functional category of genes with the background (see Figure 2C). The intensity of red colour over yellow colour in the normalized GERP-DAF matrix and coding GERP matrix for a functional category shows the extent of over-representation in respective bins over the genome-wide background. We ask whether the GERP-DAF matrix of a functional category is different from that of the background, and assess the statistical significance of this using a chi-square test with 8 degrees of freedom. Any functional category with chi square p-value < 0.05 without Bonferroni correction relative to the genome-wide distribution was considered significantly different. The analyses were repeated using Bonferroni correction for multiple testing. Most of the functional categories (>85%) that were significant in at least three out of four populations were also significant (p-value < 0.05) in at least one population after Bonferroni correction. The patterns were broadly consistent for different choices of boundary of the bins in the GERP-DAF matrices (data not shown).

### Clustering functional categories

In order to summarize our results, we decided to cluster the significant functional categories. We computed two sets of pair-wise Euclidean distances of the functional categories based on their extent of over-representation in the bins of 3 × 1 coding GERP matrix (3 elements) and 3 × 3 divergence-DAF matrix (9 elements) respectively. Let the distance matrix based on the 3 × 1 coding GERP matrix and the 3 × 3 divergence-DAF matrix be D1 and D2 respectively. The final matrix, D, satisfies the following boundary conditions: (i) The closeness (C) between pairs of functional categories depends on D1 when other factors remain unchanged. (ii) Since the distances in D1 and D2 are not directly comparable (non-metric) as they are in different scales, D should not depend on the choice of scale for D1 or D2 (iii) If two functional categories are very similar in both their coding GERP matrix and divergence-DAF matrix (*i.e*. have small distances in D1 and D2), they have a small distance in D. If, on the other hand, a pair of functional categories is dissimilar both in coding GERP matrix and GERP-DAF matrix, they will have a large distance in D. We obtained a combined distance matrix, D, by multiplying pair-wise distances between functional categories in D1 and D2 and clustered the functional categories using unsupervised hierarchical complete linkage clustering based on the matrix D. Although clustering could be performed using several other methods, the clustering described above satisfied all boundary conditions and the result was consistent with our biological understanding that similar functional categories were clustered together.

### Structural implication of the SNPs

DNA-binding domains associated with nonsynonymous SNPs in Cluster I were identified using the Superfamily [47] and DBD [48] databases. For these domains, homologus structures co-crystallized with DNA were identified from the Protein Data Bank with ≥ 35% identity. In the homologus structure, the area in contact with DNA for the residue corresponding to the allelic position was calculated using NACCESS (Hubbard and Thornton 1993).

References for publicly available tools used in the study are mentioned in the text and can be obtained from their respective sources; data used in this study and scripts for the analyses described here can be obtained from the authors upon request.

## Authors' contributions

SD and SAT conceived of the study, and participated in its design and coordination. SD carried out the experiments. SD, NLB and SAT analysed the data. SD, NLB and SAT wrote the paper. All authors read and approved the final manuscript.

## Supplementary Material

Additional file 1 Allele frequency distribution. (A) The ancestral allele of a single nucleotide polymorphism (SNP) was determined by first aligning the orthologous protein sequences between human and chimpanzee and aligning the cDNA sequences accordingly. (B) Distribution of SNPs with derived allele frequency (DAF) for Chinese Han (CHB), Japanese (JPT), Yoruba (YRI) and European (CEU) populations for all coding SNPs (top) and only non-synonymous SNPs (bottom). (C) Plot of DAF of SNPs between pairs of populations. Click here for file

Additional file 2 GERP score distribution. (A) The phylogenetic tree of the mammalian genomes used to calculate divergence rate are shown. (B) Distribution of GERP score for coding SNP and non-SNP bases. The pattern is shown for synonymous and non-synonymous base positions. The coding SNP positions are more divergent than the coding non-SNP positions. Click here for file

Additional file 3 Divergence-DAF distribution. Genome-wide distribution of divergence and DAF for both synonymous and nonsynonymous SNPs. Derived allele frequency (DAF) of SNPs in the dataset was binned into low ∈ (0.05, 0.33), intermediate ∈ (0.33, 0.67) and high ∈ (0.67, 0.95). Divergence (GERP score) was binned into conserved (< -1), intermediate ∈ (-1, 1) and divergent (>1). Intensity of blue colour is proportional to the proportion of SNPs in them. The genome-wide distribution shows that most SNPs have low DAF, especially for conserved positions. Click here for file

Additional file 4 Functional categories deemed significant in the BaseDiver analysis. GERP-DAF distribution of SNPs in (A) the GO Biological Process and (B) the GO Molecular Function categories that were significantly different from the genome-wide background in at least three out of the four HapMap populations. Bin-sizes in the matrices are the same as in Figure 3 in the main manuscript. Extent of overrepresentation is indicated by the intensity of red colour. The intensity of blue colour in adjacent circles indicates statistical significance in a Chi square test (without Bonferroni correction) for functional categories in each HapMap population. Click here for file

Additional file 5 List of common nsSNPs with large inter-population differences in DAFs. A list of all common (minor allele frequency>0.05) nonsynonymous SNPs that have large difference (>0.75) in DAF between two HapMap populations are shown. Click here for file

Additional file 6 BaseDiver Analysis of Panther Categories. BaseDiver analysis was repeated using functional categories based on the PANTHER functional classification. The results were compared with those using GO classification. Click here for file

Additional file 7 Estimation of Ascertainment Bias. The impact of ascertainment bias in the HapMap data on our analysis was analysed. Click here for file
