# Supplementary material for: Patterns of evolutionary constraints on genes in humans
Source: BMC Evol Biol. 2008 Oct 7;8:275. doi: 10.1186/1471-2148-8-275 (PMC2587479; doi:10.1186/1471-2148-8-275)
Supplement: Additional file 1 — Allele frequency distribution. (A) The ancestral allele of a single nucleotide polymorphism (SNP) was determined by first aligning the orthologous protein sequences between human and chimpanzee and aligning the cDNA sequences accordingly. (B) Distribution of SNPs with derived allele frequency (DAF) for Chinese Han (CHB), Japanese (JPT), Yoruba (YRI) and European (CEU) populations for all coding SNPs (top) and only non-synonymous SNPs (bottom). (C) Plot of DAF of SNPs between pairs of populations. [file 1471-2148-8-275-S1.pdf]

Additional File 1: Allele frequency distribution

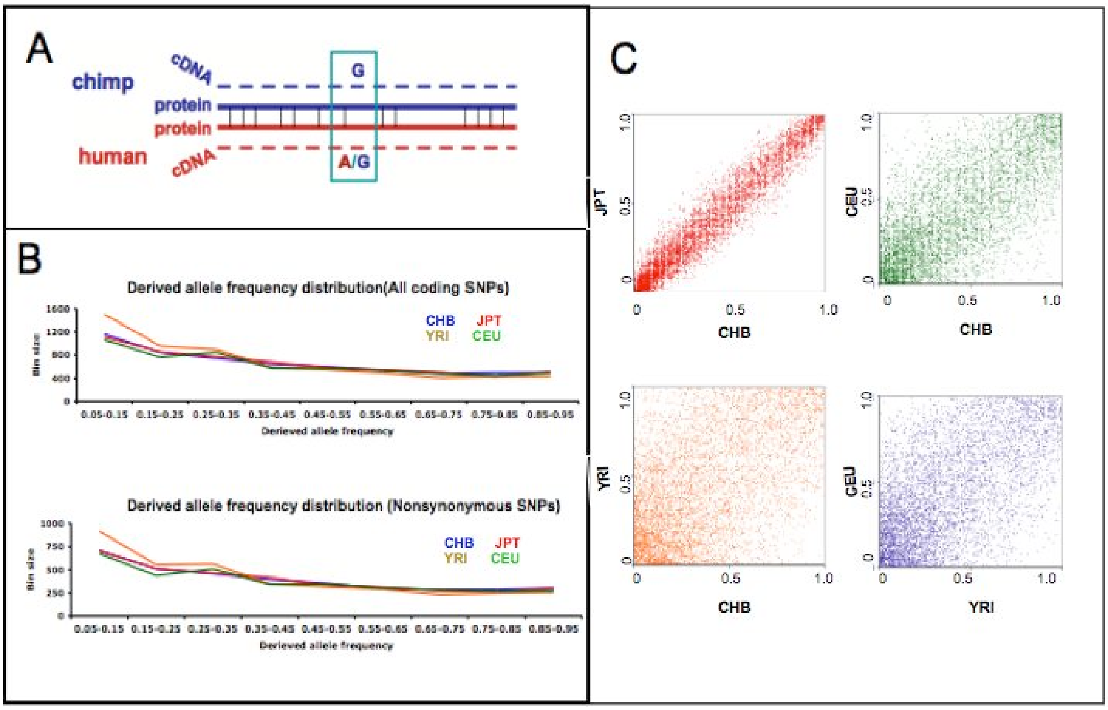

Additional File 1: (A) The ancestral allele of a single nucleotide polymorphism (SNP) was determined by first aligning the orthologous protein sequences between human and chimpanzee and aligning the cDNA sequences accordingly. (B) Distribution of SNPs with derived allele frequency (DAF) for Chinese Han (CHB), Japanese (JPT), Yoruba (YRI) and European (CEU) populations for all coding SNPs (top) and only non-synonymous SNPs (bottom). (C) Plot of DAF of SNPs between pairs of populations. DAF of CHB and JPT are highly similar.
