# Supplementary material for: Patterns of evolutionary constraints on genes in humans
Source: BMC Evol Biol. 2008 Oct 7;8:275. doi: 10.1186/1471-2148-8-275 (PMC2587479; doi:10.1186/1471-2148-8-275)
Supplement: Additional file 2 — GERP score distribution. (A) The phylogenetic tree of the mammalian genomes used to calculate divergence rate are shown. (B) Distribution of GERP score for coding SNP and non-SNP bases. The pattern is shown for synonymous and non-synonymous base positions. The coding SNP positions are more divergent than the coding non-SNP positions. [file 1471-2148-8-275-S2.pdf]

Additional File 2: GERP score distribution

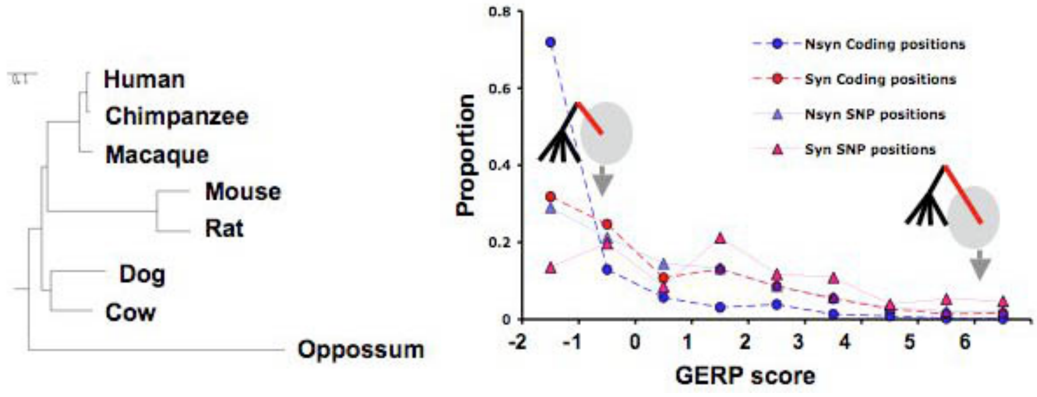

Additional File 2: (A) The phylogenetic tree of the mammalian genomes used to calculate divergence rate. (B) Distribution of GERP score for coding SNP and non-SNP bases. The pattern is shown for synonymous and non-synonymous base positions. The coding SNP positions are more divergent than the coding non-SNP positions.
