# Supplementary material for: Patterns of evolutionary constraints on genes in humans
Source: BMC Evol Biol. 2008 Oct 7;8:275. doi: 10.1186/1471-2148-8-275 (PMC2587479; doi:10.1186/1471-2148-8-275)
Supplement: Additional file 3 — Divergence-DAF distribution. Genome-wide distribution of divergence and DAF for both synonymous and nonsynonymous SNPs. Derived allele frequency (DAF) of SNPs in the dataset was binned into low ∈ (0.05, 0.33), intermediate ∈ (0.33, 0.67) and high ∈ (0.67, 0.95). Divergence (GERP score) was binned into conserved (< -1), intermediate ∈ (-1, 1) and divergent (>1). Intensity of blue colour is proportional to the proportion of SNPs in them. The genome-wide distribution shows that most SNPs have low DAF, especially for conserved positions. [file 1471-2148-8-275-S3.pdf]

Additional File 3: Divergence-DAF distribution

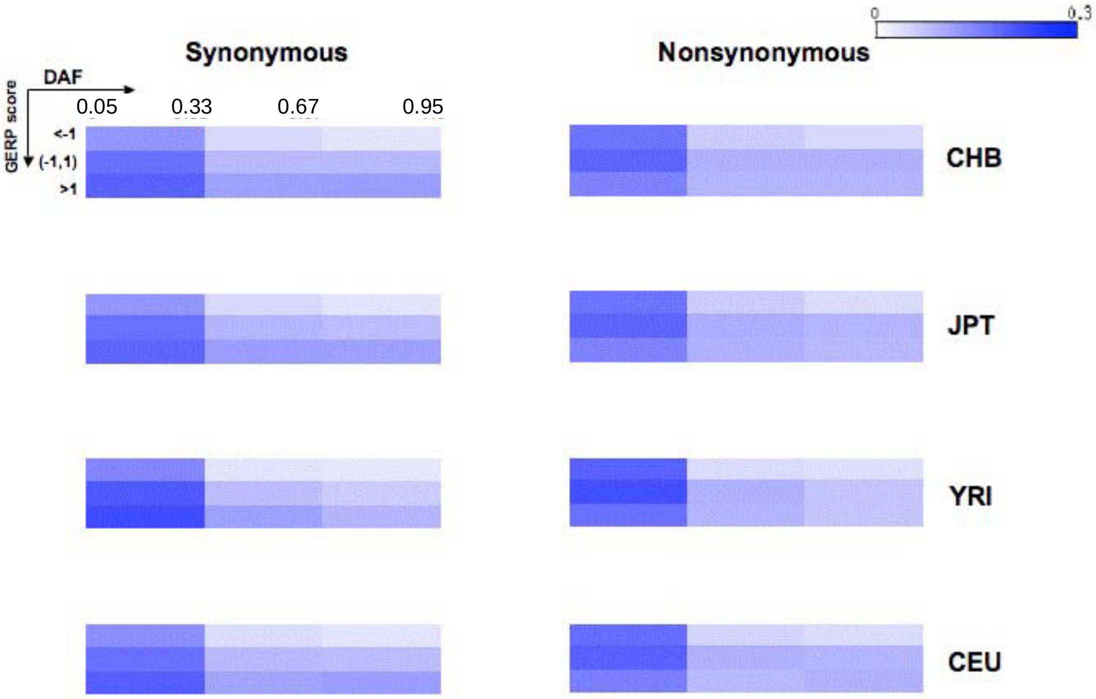

Additional File 3: Genome-wide distribution of divergence and DAF for both synonymous and nonsynonymous SNPs. Derived allele frequency (DAF) of SNPs in the dataset was binned into low ∈ (0.05, 0.33), intermediate ∈ (0.33, 0.67) and high ∈ (0.67, 0.95). Divergence (GERP score) was binned into conserved (< -1), intermediate ∈ (-1, 1) and divergent (>1). Intensity of blue colour is proportional to the proportion of SNPs in them. The genome-wide distribution shows that most SNPs have low DAF, especially for conserved positions. At divergent positions there are more SNPs with intermediate or high DAF.
