# Supplementary material for: Patterns of evolutionary constraints on genes in humans
Source: BMC Evol Biol. 2008 Oct 7;8:275. doi: 10.1186/1471-2148-8-275 (PMC2587479; doi:10.1186/1471-2148-8-275)
Supplement: Additional file 4 — Functional categories deemed significant in the BaseDiver analysis. GERP-DAF distribution of SNPs in (A) the GO Biological Process and (B) the GO Molecular Function categories that were significantly different from the genome-wide background in at least three out of the four HapMap populations. Bin-sizes in the matrices are the same as in Figure 3 in the main manuscript. Extent of overrepresentation is indicated by the intensity of red colour. The intensity of blue colour in adjacent circles indicates statistical significance in a Chi square test (without Bonferroni correction) for functional categories in each HapMap population. [file 1471-2148-8-275-S4.pdf]

Additional File 4: Functional categories deemed significant in the BaseDiver analysis

A. GO Biological Process categories

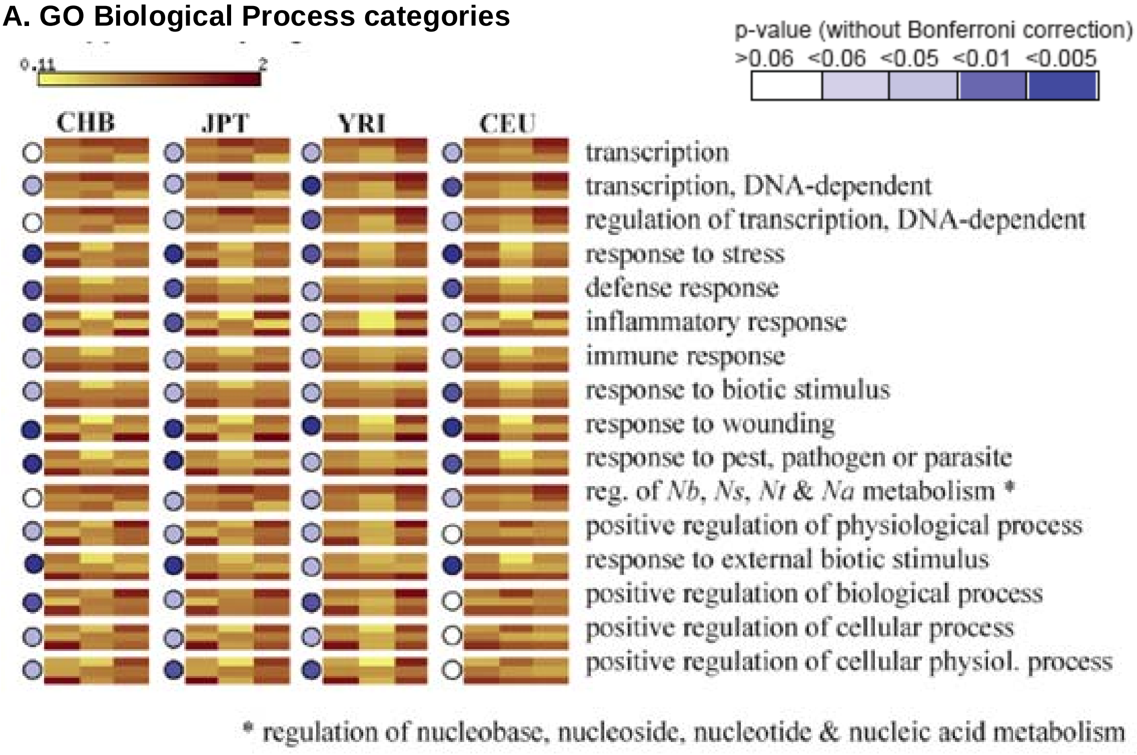

B. GO Molecular Function categories

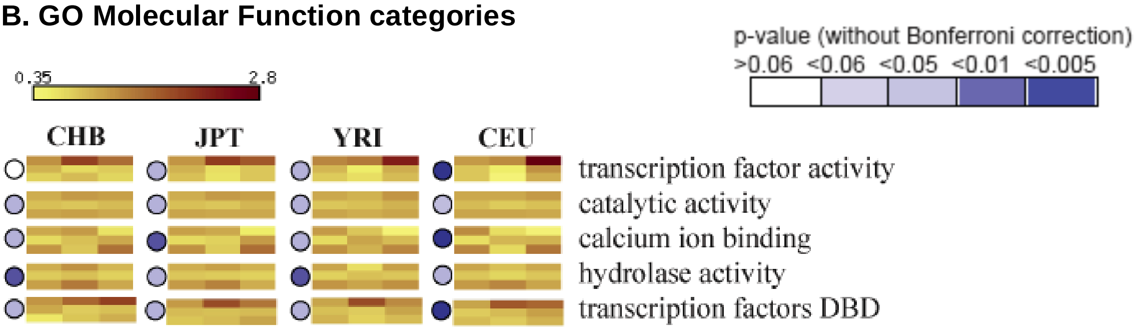

Additional File 4. GERP-DAF distribution of SNPs in (A) the GO Biological Process and (B) the GO Molecular Function categories that were significantly different from the genome-wide background in at least three out of the four HapMap populations. Bin-sizes in the matrices are the same as in Figure 3 in the main manuscript. Extent of overrepresentation is indicated by the intensity of red colour. The intensity of blue colour in adjacent circles indicates statistical significance in a Chi square test (without Bonferroni correction) for functional categories in each HapMap population.
