# Supplementary material for: Patterns of evolutionary constraints on genes in humans
Source: BMC Evol Biol. 2008 Oct 7;8:275. doi: 10.1186/1471-2148-8-275 (PMC2587479; doi:10.1186/1471-2148-8-275)
Supplement: Additional file 6 — BaseDiver Analysis of Panther Categories. BaseDiver analysis was repeated using functional categories based on the PANTHER functional classification. The results were compared with those using GO classification. [file 1471-2148-8-275-S6.pdf]

Additional File 6: BaseDiver Analysis of Panther Categories

To check that our conclusions are not dependent on the particular functional categories defined by the GO database, we repeated the BaseDiver analyses on *Biological Processes* and *Molecular Function* using the Panther (***P**rotein **A**nalysis **T**hrough **E**volutionary **R**elationships*) scheme (Mi *et al.* 2004). Panther uses direct experimental evidence as well as evolutionary relationships to assign functionality. Some GO categories have a one-to-one correspondence with Panther categories while for others no one-to-one mapping is possible. For example, *Catalytic Activity* in the GO classification overlaps with *Hydrolase, Kinase, Ligase, Isomerase, Lyase* in the Panther classification. The overlap of categories between the two classifications is shown in Figure AF6.1.

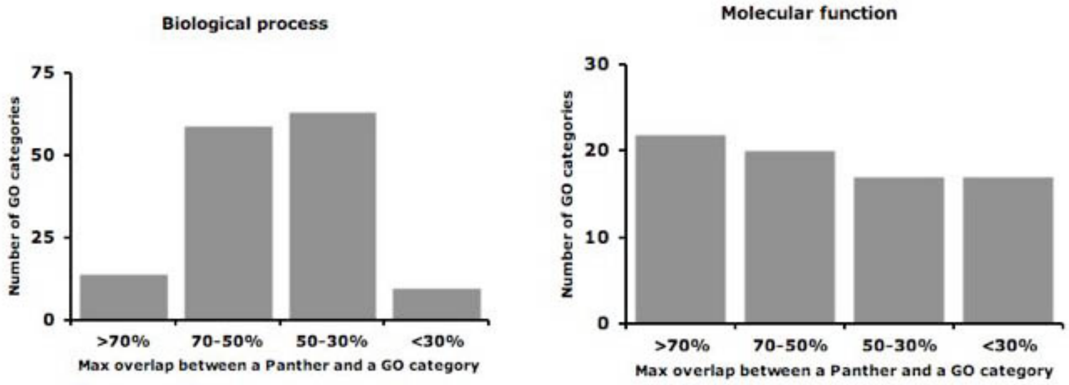

Figure AF6.1: Fraction of the number of genes in a single GO category also in a single Panther category. The maximum overlap is taken, and can vary between 0 – 100%. The number of GO categories is shown against extent of maximum overlap with a Panther category. The analysis is restricted to the GO categories considered for BaseDiver analysis *i.e.* those with >100 SNPs.

Despite the partial overlap between the GO and Panther classifications, the signature of evolutionary constraints on equivalent categories shows a similar pattern. The categories with a significant deviation from the genomic background in the BaseDiver analysis of GO functional categories are shown in Figure 3 of the manuscript. Many of these have equivalent Panther categories that show a consistent pattern of constraints as compared to the genome-wide divergence-DAF distribution, as described below.

Cluster I: Several GO transcriptional regulatory-related categories were found to show high frequency derived alleles in human when that base was otherwise highly

constrained in mammals. In the Panther-based analysis the category called *Transcription factors* also shows a similar pattern (Figure AF6.2). The overlap of this Panther category with equivalent GO categories is high (*Transcription factor activity:* 83%*, Transcription, DNA dependent:* 68% and *Regulation of nucleobase, nucleoside, nucleotide and nucleic acid metabolism: 65% etc)*. In the Panther-based analysis the signature is statistically significant ($p<0.05$) in all four populations, even after Bonferroni correction. This provides strong support for lineage specific changes on transcription factors in humans.

Cluster II: Immunity and Defense in Panther has a moderate overlap with several GO categories - Defense Response (51%), Immune Response (54%), Response to Stress (39%), Response to wounding (55%), Response to biotic stimulus (49%) and Response to pest, pathogen or parasite (56%). The divergence-DAF distribution of SNPs in Defense and Immunity in Panther shows patterns partially consistent with that observed in the GO-based classification.

Cluster III: Several GO categories related to olfactory perception such as *Perception of smell* and *Olfactory receptors* are under reduced constraints. These GO categories mostly overlap with the categories *Receptors* and *Sensory perception* in Panther classification. The overlap between *Receptor* in the Panther classification and *Olfactory receptor activity* in GO classification has 53% overlap, while the overlap between *Sensory perception* according to the GO and Panther classifications is 36%. While the number of SNPs in the category of *Sensory perception* in the Panther classification was too small for statistical significance, *Receptors* show significant reduced constraints. This is probably due to the large number of olfactory receptors in this category.

Cluster IV: The functional category of *Hydrolase activity* also shows the signature of negative selection as observed in GO, although it is not statistically significant across populations. The overlap between *Hydrolase activity* categories according to the GO and Panther classifications is 32%.

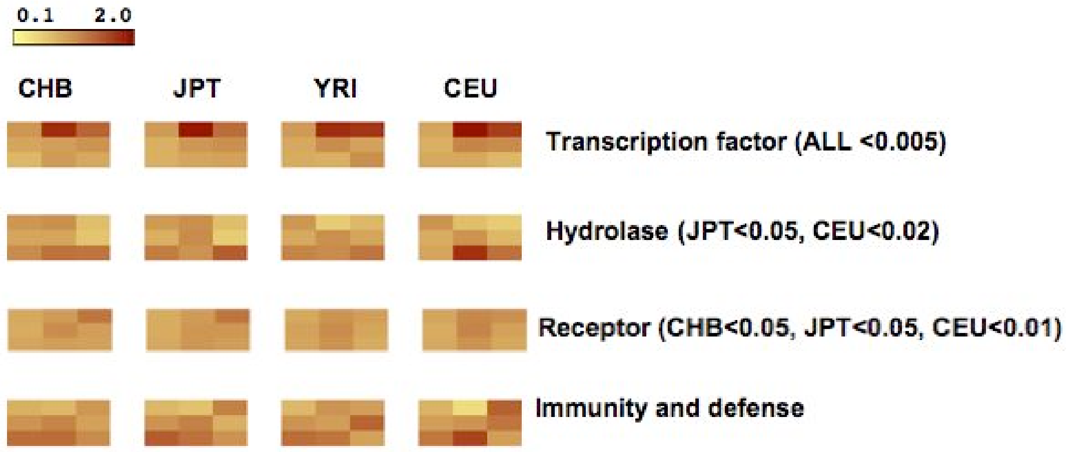

Figure AF6.2: Divergence-DAF distributions relative to the genome-wide background for Panther categories equivalent to the significant GO categories. The divergence-DAF enrichments are similar using both classification schemes.

This analysis shows that most categories are in good agreement across the GO and Panther classification schemes, and that the patterns of evolutionary constraints are independent of the functional classification used.
