# Supplementary material for: Patterns of evolutionary constraints on genes in humans
Source: BMC Evol Biol. 2008 Oct 7;8:275. doi: 10.1186/1471-2148-8-275 (PMC2587479; doi:10.1186/1471-2148-8-275)
Supplement: Additional file 7 — Estimation of Ascertainment Bias. The impact of ascertainment bias in the HapMap data on our analysis was analysed. [file 1471-2148-8-275-S7.pdf]

Additional File 7: Estimation of Ascertainment Bias

Ascertainment bias arises due to non-random sampling during the process of SNP discovery employed by large-scale SNP genotyping studies. It results in missing rare SNPs, and affects analyses that depend on site-frequency spectrum (e.g. nucleotide diversity, Tajima's D, F_{st} and linkage disequilibrium). HapMap data is also affected by ascertainment bias, and rare SNPs may go undiscovered (Clark *et al*. 2005). However, as Clark and colleagues point out, each individual (common) SNP that is examined in the large sample may be accurately measured with respect to its frequency, heterogeneity among population and linkage disequilibrium.

There are three solutions to avoid ascertainment bias. First, restrict an analysis only to common SNPs (minor allele frequency >0.05), and therefore bypass the complications with identifications of rare SNPs (minor allele frequency <0.05) altogether. Second, use correction for ascertainment bias, which involves estimation of probability of discovering SNPs in a given region. Clark and colleagues point that ascertainment correction for individual genes may be problematic and discrepancies persist even after correction. Third, use of complete re-sequencing data, which has no ascertainment bias. Unfortunately complete re-sequencing based high quality polymorphism data is yet to be available.

At this end, we preferred to use the first strategy and restricted ourselves to common nonsynonymous SNPs only. To estimate the extent of bias introduced by the use of HapMap data, we did two control analyses. First, we downloaded files corresponding to Perlegen *SNP data* and dbSNP mapping from Perlegen Genome Browser V2. (http://genome.perlegen.com/browser/download.html). We identified the set of 2238 nonsynonymous SNPs that are genotyped by both HapMap and Perlegen for three similar population-pairs (HapMap:CEU / Perlegen:Eur, HapMap:CHB / Perlegen:CHN and HapMap:YRI / Perlegen:AFR). Perlegen *SNP data* and dbSNP mapping files were downloaded from (http://genome.perlegen.com/browser/download.html). A very good correlation of allele frequencies between the two studies was observed (Figure AF7.1).

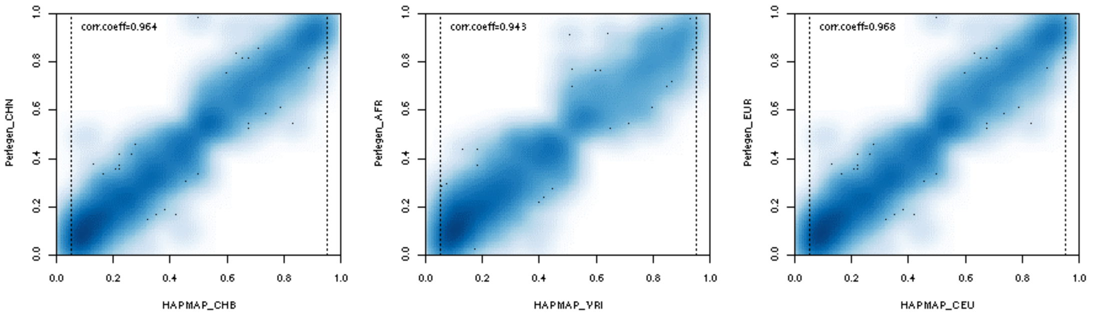

Figure AF7.1: Correlation of allele frequency between three similar HapMap-Prelegen population-pairs (HapMap:CEU / Perlegen:Eur, HapMap:CHB / Perlegen:CHN and HapMap:YRI / Perlegen:AFR). Intensity of blue colour shows density of points. Pearson Correlation coeffient is shown at top right corner. Data is shown for common SNPs in HapMap (minor allele frequency>0.05) only. Vertical dotted lines in each panel corresponds to DAF=0.05 and DAF=0.95.

Next, we compared the density of common noncoding SNPs between our dataset and those in completely re-sequenced ENCODE regions. Data for re-sequenced ENCODE regions were collected from UCSC Genome Browser (http://genome.ucsc.edu/). 95 genes were common between our dataset and re-sequenced ENCODE regions. In ENCODE analysis, there were 429 nonsynonymous SNPs, of which only 195 had minor allele frequency>0.05, and also GERP score available. In the same set of proteins we found 177 SNPs in our analysis. This comparison shows that we have probably captured a majority of the non-synonymous common SNPs. Our finding is not surprising because HapMap analysis aimed to cover majority of the common non-synonymous SNPs.

These two analyses suggest that effects of ascertainment bias in our study are probably low.
